# Sedentary Behavior, Brain-Derived Neurotrophic Factor (BDNF), and Brain Structure in Midlife: A Brain MRI Study

**DOI:** 10.1093/geroni/igaa057.3264

**Published:** 2020-12-16

**Authors:** Xuan Zhang, Denise Gaughan, Chengxuan Qiu, Osorio Meirelles, Lenore Launer

**Affiliations:** 1 National Institute on Aging, Bethesda, Maryland, United States; 2 Karolinska Institutet, Solna, Sweden

## Abstract

Long sedentary time (ST) is associated with poor brain health but the underlying mechanisms are unclear. Studies suggest exercise increases BDNF levels, and that low BDNF levels are associated with cognitive impairment. Limited population-based studies have examined associations among sedentary behavior (SB), BDNF, and brain structures. Here we explore the mediation and interaction effect of BDNF in the association of SB to brain measures. We included 612 participants from the MRI sub-study of the Coronary Artery Risk Development in Young Adults who had plasma BDNF and SB data at the Year 25 examination. SB was estimated by self-reported average ST hours/day spent sitting while watching television, using computers, and riding transportation. Outcome measures were total and selected brain volumes in cubic centimeters (cc). ST was categorized into quartiles. We used general linear regression to examine the following associations, adjusting for age, sex, race, and intracranial volume: Interactions between BDNF and ST on MRI; ST and MRI; ST and BDNF; BDNF and MRI; and ST, BDNF, and MRI. People in the upper 25%ile ST (>8.4 hours/day) had a decreased TB volume of 12.2 cc (p=0.01) compared to the lower 25%ile (<4.3 hours/day). Neither ST nor brain measures were associated with BDNF (p>0.05). Instead, BDNF interacted with ST for TB and WM (p < 0.03): The difference of brain volumes between the upper and lower 25%ile decreased with increasing BDNF levels. Accordingly, higher BDNF levels may protect brain function in the middle-aged and potentially older populations with a sedentary lifestyle.

